# Medical Clinic for the Session of 1860

**Published:** 1860-09

**Authors:** 


### ARTICLE III.

*Medical Clinic for the Session of* 1860. Service of DR. J. G. WESTMORELAND.

May 9.—A colored boy, aged 25 years, the subject of Rheumatism for two weeks—has suffered with a similar attack previously.

This is one of the cases usually denominated Nervous-Rheumatism, and there is in the constitution of the patient, evidently, a predisposition or tendency to such attacks—a rheumatic diathesis.

In the two varieties of Rheumatism, nervous and articular, the symptoms in some respects differ widely from each other, particularly in respect to the parts of the body in which we find evidences of local disturbance, and in the character of such manifestations. While the pain in nervous rheumatism is referred to the whole length, sometimes, of the limb, or between the joints only, as well as in the joints themselves, articular rheumatism, as the term implies, affects the joints almost exclusively; and while in the former variety none of the symptoms of ordinary inflammation usually exist, such as swelling, redness, heat, &c., in the latter these are almost always present. So widely and constantly do these local symptoms differ in the two varieties, that they would reasonably be referred to different primary pathological conditions, but for the light thrown upon the pathology by experience in the treatment. Indeed, without the knowledge of pathology, derived from the successful application of remedies, whose proportions are well known, the primary disturbance, not only of the two varieties of rheumatism, but of other very common diseases would not be understood.

Then it is in this, as it has been in other diseases, particularly mallarious fever, the empyrical, or, perhaps, more properly speaking; experimental prescriptions made from time to time, has finally led to the discovery of the agent or mode of treatment which changes the pathological condition —counteracts the primary disturbance, cures the disease. Now, whatever organ or system of the body is known to be influenced by such agents or plans of treatment, is that which we conclude is primarily disturbed. If, for example, remittent fever, by the use of cinchona, is relieved, arrested, cured, (as is known to be the case), and if it also be ascertained that the important, prominent action of this article is exerted upon the nervous system, may we not infer that this system is primarily diseased or deranged in this fever, particularly when the usual premonitory, as well as permanent symptoms of the disease, are readily accounted for under this pathological view?

Now, gentlemen, quinine is not more certain to arrest the progress of malarial fever than it is to allay the symptoms of rheumatism. Not only in those cases—such as the one before us—in which there is evidently nervous derangement, and which make one of the varieties mentioned, but even more perfectly does it control *acute articular rheumatism.* That case exhibiting swollen, hot, red and painful joints, with general fever, is the one in which quinine is found especially useful. At the same time, it is known that this remedy has no direct influence upon any of the organs or tissues in which these local symptoms are found. While we have the usual symptoms of ordinary inflammation in acute articular rheumatism, its migratory character, and other characteristics of the disease prove the origin to be something more than the cause of ordinary inflammation.

From various considerations connected with the symptoms and tendencies of the malady, as well as the success of an agent whose action is exerted almost exclusively upon the nervous system, we conclude that the primary disturbance of rheumatism must exist in that system; particularly since the peculiarities are more explicable under this pathological view.

In chronic rheumatism, we do not usually realize such prompt benfit from the use of quenine, whether nervous or articular, and a satisfactory explanation exists in the fact that the *results* of rheumatic inflammation upon the tissues in which it is located, are often the cause of symptoms which are found under such circumstances. These results, which by deposits and otherwise, destroy the usual elasticity, smoothness and freedom of motion those parts naturally possess, which are the usual location of such disease. Such exist, not from a dependence upon the primary cause, very often, but from the local disease, or results of rheumatism, hence are not under the control entirely of such means as are effectual in arresting acute rheumatism.

In the treatment of rheumatism, as in the management of many other affections, we sometimes fail of success, not because we fail to use the proper agents, but because the remedy is not used in proper quantity, or at the proper time. Hence, practitioners become discouraged, in the use of proper medicinal agents. Quinine, in the treatment of rheumatism as in fever, should be given in quantities sufficient to impress the nervous system fully.

In the first suggestion I ever saw of the use of the remedy for this purpose, (which, by the bye, was in a Medical Journal, from the pen of a practitioner in the country,) the quantity advised was five grains every night, combined with two grains of opium. This dose is not sufficient, however, to effect all the good that may be obtained from quinine in rheumatism. In many cases we have rapid improvement from its use in this quantity, even with such long intervals, but there are other cases which do not yield to this course. In order to insure the tonic or invigorating influence of it upon the nervous centre, sufficient to counteract the disease, the amount of fifteen or twenty, and sometimes thirty grains is required.

In the case before us, I make the following prescription:

℞—Quinia sulph., grs. xx.
Pulv. Ipecac. et opii. grs. x.
Misce et fiat chart. no. ii.

Give one at bed-time to-night, and one to-morrow morning.

